# Live‐fast‐die‐young: Carryover effects of heatwave‐exposed adult urchins on the development of the next generation

**DOI:** 10.1111/gcb.16339

**Published:** 2022-08-03

**Authors:** Jay J. Minuti, Maria Byrne, Hamish Campbell, Deevesh A. Hemraj, Bayden D. Russell

**Affiliations:** ^1^ The Swire Institute of Marine Science and Area of Ecology and Biodiversity, School of Biological Sciences The University of Hong Kong Hong Kong SAR China; ^2^ Institute for Climate and Carbon Neutrality The University of Hong Kong Hong Kong SAR China; ^3^ School of Life and Environmental Sciences The University of Sydney Sydney New South Wales Australia; ^4^ The Dove Marine Laboratory School of Natural and Environmental Sciences Newcastle University Newcastle‐upon‐Tyne UK

**Keywords:** carryover effects, climate change, *Heliocidaris erythrogramma*, marine heatwave, sea urchin, thermal physiology

## Abstract

With rising ocean temperatures, extreme weather events such as marine heatwaves (MHWs) are increasing in frequency and duration, pushing marine life beyond their physiological limits. The potential to respond to extreme conditions through physiological acclimatization, and pass on resistance to the next generation, fundamentally depends on the capacity of an organism to cope within their thermal tolerance limits. To elucidate whether heat conditioning of parents could benefit offspring development, we exposed adult sea urchins (*Heliocidaris erythrogramma*) to ambient summer (23°C), moderate (25°C) or strong (26°C) MHW conditions for 10 days. Offspring were then reared at constant temperature along a thermal gradient (22–28°C) and development was tracked to the 14‐day juvenile stage. Progeny from the MHW‐conditioned adults developed through to metamorphosis faster than those of ambient conditioned parents, with most individuals from the moderate and strong heatwaves developing to the larval stage across all temperatures. In contrast, the majority of offspring from the control summer temperature died before metamorphosis at temperatures above 25°C (moderate MHW). Juveniles produced from the strong MHW‐conditioned adults were also larger across all temperatures, with the largest juveniles in the 26°C treatment. In contrast, the smallest juveniles were from control (current‐day summer) parents (and reared at 22 and 25°C). Surprisingly, initial survival was higher in the progeny of MHW exposed parents, even at temperatures hotter than predicted MHWs (28°C). Importantly, however, there was substantial mortality of juveniles from the strong MHW parents by day 14. Therefore, while carryover effects of parental conditioning to MHWs resulted in faster growing, larger progeny, this benefit will only persist beyond the more sensitive juvenile stage and enhance survival if conditions return promptly to normal seasonal temperatures within current thermal tolerance limits.

## Graphical abstract

Marine heatwaves (MHWs) can have devastating effects on marine ecosystems by driving widespread mortality of organisms, but individuals which survive the thermal stress may be able to pass heat tolerance to their offspring. We show that MHW‐conditioned sea urchins produce faster‐growing, larger and more heat tolerant offspring. If heatwaves continued too long, however, there was high mortality even in progeny from heatwave‐exposed parents. Therefore, the beneficial carryover effects of parental conditioning to MHWs will only persist beyond the more sensitive juvenile stage, and enhance survival, if MHWs are not extended and temperatures promptly return to normal.

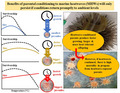

## 1 INTRODUCTION

Global ocean temperatures are increasing as warming trends accelerate under anthropogenic forcing, causing considerable risk to marine communities (Gattuso et al., 2015; Hughes et al., 2017; Wernberg et al., 2011). In parallel, marine ecosystems are being exposed to acute and extreme temperature increase in heat spikes and marine heatwaves (Hobday et al., 2018; Smale et al., 2019; Wernberg et al., 2016). As a result, organisms are exposed to sudden and rapid increases in water temperature, requiring rapid physiological acclimatization. Such physiological adjustments often involve high energy demands and simultaneously cause direct costs to the organism, such as reduced reproductive output, nonadaptive metabolic depression or mortality (Chandrapavan et al., 2019; Hemraj et al., 2020; Piatt et al., 2020; Wild et al., 2019) as well as transgenerational impacts such as offspring physiological stress and lower maternal investment in offspring leading to reduced size and survival of offspring (Gibbs et al., 2021a, 2021b; Harianto et al., 2021; Leicht & Seppälä, 2019).

The impact of increased temperature on marine ectotherms depends on their physiological capacity to adjust to change and cope within thermal tolerance limits (Babcock et al., 2019; Hardy & Byrne, 2014; Minuti & Russell, 2020; Nguyen et al., 2011; Pörtner, 2012). For example, larvae which experience a wide temperature range during their dispersal phase tend to have relatively higher thermal tolerance limits, giving them greater options for habitat relocation if populations need to escape extreme thermal stress (Byrne et al., 2022; Sorte et al., 2018). In contrast to seasonal temperature variation or gradual warming, MHWs exert a sudden, strong thermal pressure on marine organisms that can quickly exceed their tolerance limits and reduce their ability to acclimate. Under such conditions, larvae will rely on their parents' ability not only to survive the extreme events, but also to pass on protective mechanisms which will reduce their stress sensitivity during development and increase their chance of survival under continued environmental stress (Karelitz, Lamare, Mos, et al., 2019; Suckling et al., 2014).

The rate of larval development and planktonic larval duration have positive and negative relationships with temperature, respectively, up to a limit when deleterious effects occur due to stress and mortality (Byrne, 2011). In sea urchins, early embryos are often tolerant of increased temperature (Byrne et al., 2009, 2011; Hardy et al., 2014; O'Donnell et al., 2009) potentially due to the presence of protective maternal factors that diminish throughout development with mortality increasing at the later stages (Hamdoun & Epel, 2007). Indeed, maternal provisioning plays a key role in sea urchin larval ontogenetic change whereby larvae are provisioned with lipids and protein to fuel development into functional feeding larvae. If larvae are maternally supplied with protective mechanisms, survival is increased in the next generation, allowing populations to persist (Byrne et al., 2008; Byrne & Sewell, 2019; Sewell, 2005).

In this study, we tested whether exposure of sea urchin (*Heliocidaris erythrogramma*) parents to different intensities of MHWs results in protective carryover effects in the development for offspring exposed a range of temperatures that they could be exposed to in the environment*. H. erythrogramma* is abundant in intertidal and shallow subtidal habitats along the temperate coast of Australia (Keesing, 2001, 2020; Smale & Wernberg, 2014; Vanderklift & Kendrick, 2004) where it plays a strong role in structuring macroalgal communities (Ling et al., 2010; Wright et al., 2005). The region is a global warming hotspot experiencing a marked increase in MHWs (Babcock et al., 2019). Recent catastrophic MHWs in the region have had profound effects across diverse taxonomic groups, with adults exposed to temperatures close to and above their thermal tolerance limits (Bennett et al., 2015; Smale et al., 2017; Thompson et al., 2013, 2015; Wernberg et al., 2013), but little is known about the potential for carryover effect of parental stress for offspring. Therefore, to elucidate whether heat hardening of parents could lead to increased survival in offspring, we exposed adult *H. erythrogramma* to heatwave scenarios, as defined by Hobday et al. (2018). This species is a good model species to investigate cross‐generational carryover effects (see Byrne et al., 2019) due to its fast development to the juvenile stage (within a week) compared with the longer planktonic duration of other echinoderms (Henry et al., 1991; Williams & Anderson, 1975; Wolfe et al., 2013). We tested the hypothesis that heatwave‐exposed parents would produce offspring with increased heat tolerance to thermal stress, which would be expressed as faster developmental times and increased survival at higher temperatures. Critically, increasing the ability of offspring to cope with higher temperatures is a key mechanism for climate adaption to facilitate population persistence and safeguard local populations from decline, and local extirpation due to increasingly frequent and intense thermal anomalies that are entrained for the foreseeable future (IPCC, 2021).

## 2 METHODOLOGY

### 2.1 Collection of adult urchins

*Heliocidaris erythrogramma* spawns from November to April and were collected by snorkelling in March–April 2019 during low tide at Little Bay, Sydney (33°58′S, 151°14′E), a shallow (0.5 m) open ocean site where this species is found in shallow‐subtidal boulder habitat. The urchins were transported immediately to the Sydney Institute of Marine Science, where they were placed in 50 L aquaria supplied with a natural flow‐through filtered seawater (FSW, 20 μm) system at ambient temperature (~23°C). They were maintained in these holding aquaria for 5 days to monitor their post collection condition and fed daily with kelp *Ecklonia radiata* and fucoid *Sargassum* sp. collected fresh daily from near the research station.

### 2.2 Adult heatwave conditioning

Adult urchins were exposed to two heatwave scenarios (Figure S1); the ‘moderate heatwave’ treatment was at peak 25°C and the ‘strong heatwave’ at 26°C as determined by regional data and recent classifications for naming heatwaves (MHWs; Hobday et al., 2016) with continuous flow‐through FSW (20 μm, 0.3 L min^−1^). Control treatments were maintained at 23°C throughout the adult conditioning period. After a 5‐day adjustment period in ambient conditions (23°C), individual urchins (*n* = 30 per treatment) were transferred into separate plastic aquaria (2 L, *n* = 30 replicate aquaria per treatment) in a temperature‐controlled room, with 12:12 h light:dark light regime similar to natural light conditions. Each individual was supplied with its own in‐flow pipe containing water adjusted to treatment temperatures in header tanks and the aquaria were placed in a water bath to maintain temperature stability. Replicate aquaria were provided with flow‐through seawater supplied by a thermocouple solenoid feedback system with constant monitoring and controlled by computer software. The control urchins were maintained at 23°C. For the heatwave treatments, the temperature was increased 0.5°C per day to reflect known rates of MHW onset in the region (Hobday et al., 2016; Reynolds et al., 2007) until the target heatwave temperature was reached. This temperature was held for 10 days, at which time all individuals were removed to induce spawning.

### 2.3 Adult spawning

**FIGURE 1:**
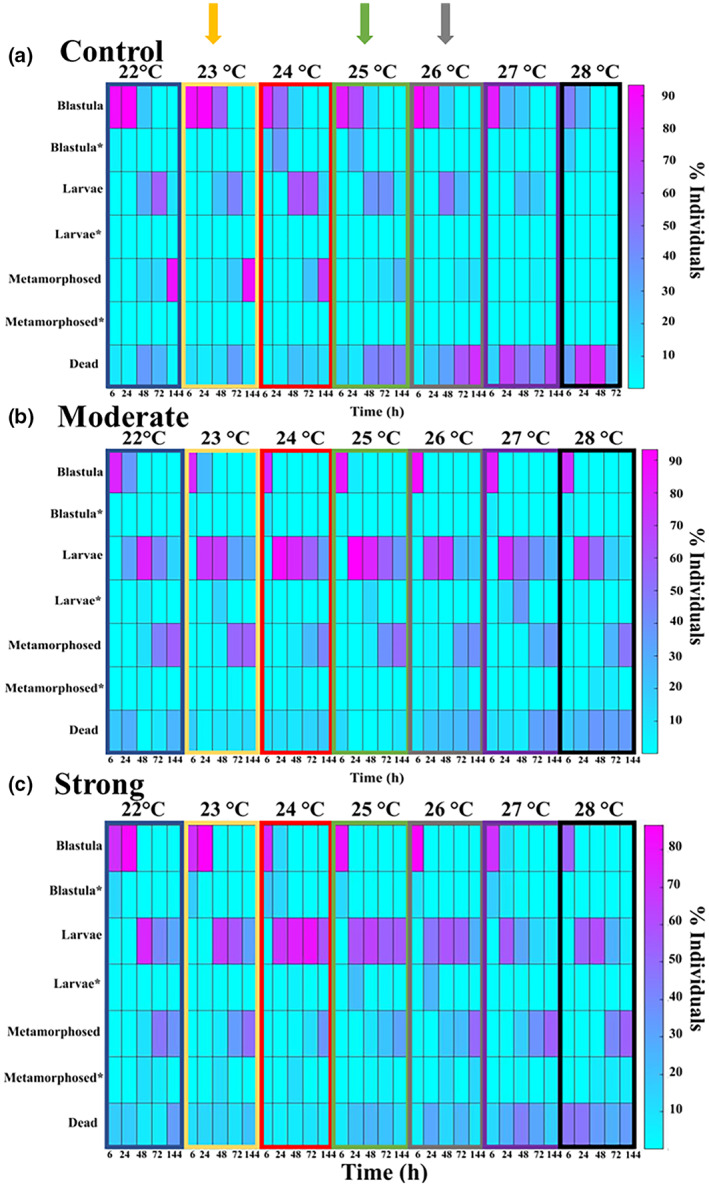
Heatmap representing the change in developmental stages of individuals reared at different temperatures and spawned in (a) control conditions, (b) moderate heatwave or (c) strong heatwave. Heatmaps show the mean percentage of individuals at each developmental stage. Individuals with abnormal development are represented by (*). Arrows above the panels represent larvae that were spawned and grown within the same treatment temperatures. Yellow, control; green, moderate heatwave; Grey, strong heatwave.

Urchins were induced to spawn on day 10 of the simulated heatwave by injecting 0.5–1 ml of 1 M KCl into the coelom. Sperm was collected directly from each male urchin using a pipette and the eggs of each female were placed in separate beakers with fresh aerated filtered seawater (FSW 1.0 μm). To ensure that egg and sperm quality were as similar as possible across treatments, gametes of the most fecund individuals as determined by the production of the abundant gametes which were examined microscopically for quality (egg shape or sperm motility) and the best gametes were used for fertilization. Eggs were pipetted into 200 ml beakers with FSW at the respective parental treatment temperature and, after pooling sperm in a Petri dish and activating with seawater, 1–3 drops of sperm were added to each beaker. After 10 min, eggs were checked microscopically to ensure fertilization success (>90%). Three independent fertilizations were done for each heatwave treatment and four for the control treatment (Figure 1). Some embryos were then reared in the temperature block (see below) and others were reared in glass beakers (with daily experimental water renewal) for respiration measurements (see below for rearing conditions).

### 2.4 Offspring rearing across a temperature gradient

Temperature treatments were created using two aluminium temperature blocks set up in parallel that allowed for 12 temperature treatments with 1°C increments across a static thermal gradient, established using hot (28°C) and cold (22°C) water inputs at either end (Figure S1). The 22–28°C range included the temperature experienced by the parents and the upper lethal temperatures for this species (Gall et al., 2021). Each block had four columns of holes to fit the glass vials used across the 12 temperatures. The eighth column was used to keep vials with water at test temperatures for water changes. Half the water (ca. 15 ml), in each vial was replaced with new FSW every 24 h to maintain dissolved oxygen (DO) levels. DO was measured with an optical DO probe (Vernier Software and Technology) and always remained above 90% saturation. The temperature of each vial was recorded daily. In all, 30 embryos from each of the fertilization replicates were placed into 30 ml glass vials and randomly placed in each row within the temperature block system (*n* = 3 vials containing 30 larvae for each treatment; control, moderate and strong heatwave treated adults). The timing of placement into the temperature block was staggered according to the timing of the adult heatwave treatment (and therefore spawning time), providing enough space in the block for all the vials.

### 2.5 Embryonic development

Developmental stage was assessed microscopically at 6 h, 24 h, 72 h and 6 days after fertilization in 15 individuals randomly sampled from each vial. Stage achieved was scored, after which the individuals were placed back into the vial. Any dead embryos were enumerated and removed from the vials. After 14 days, 15 individuals from each vial were digitally photographed and the size of each individual was recorded in mm using a compound microscope (Olympus DP70) at 20× magnification, which provides a scale bar suitable for measuring specimens using the software interface DP2‐BSW (ver.2.2).

### 2.6 Respiration rate

Juveniles (14‐day old) from the cultures reared in glass beakers in parental temperature conditions were used for metabolic rate (MO_2_) determination in these same temperatures. There were insufficient juveniles which survived to 14 days from the strong heatwave‐conditioned parents to measure respiration. In all, 30 randomly selected juveniles (from the beakers described above) were placed into 5 ml glass vials (control *n* = 18 vials, moderate *n* = 15 vials) and placed into a water bath at the corresponding heatwave temperature for measurements to take place. Oxygen concentration in each vial was recorded every 1 min for 2.5 h using an internal optical oxygen spot measuring system (Fibox). Respiration rate MO_2_ (μg L^−1^ ind^−1^ h^−1^) was then calculated using the following equation:

$\frac{{\mathrm{MO}}_{2}=\Delta O_{2}V}{N},$

where ∆O_2_ is the linear regression slope of oxygen concentration over time (μg L^−1^ h^−1^), *V* is the volume of seawater (L) and *N* is the number of juveniles in each vial. Blank chambers containing no juvenile urchins were run to account for any possible biological activity in the water (*n* = 6 blanks for the moderate treatment, *n* = 4 for the control).

### 2.7 Statistical analysis

Data were first tested for normality and homoscedasticity using a Shapiro–Wilk and Barlett's test, respectively (Table S1). The overall differences in the number of individuals from each treatment reaching each developmental stage (blastula, larvae, metamorphosed or dead) at each time point (6, 24, 48, 72 and 144 h) were analysed using two‐factor permutational MANOVAs (factors: time, treatment condition). When significant effects were detected, post hoc pairwise tests were used to determine which treatments differed. To account for inflated error rates with multiple comparisons, significance was judged with the more conservative *p* < .01. The differences in size and survival of juveniles originating from each parental heatwave treatment exposed to different temperatures (22–28°C) were analysed using a single‐factor PERMANOVA and the trends in response across the temperature range were analysed by estimating the best‐fit curve based on *R* ^2^‐value, significance of fit (*p*‐value), AIC and BIC (Table S2). Respiration rates were analysed using distance‐based permutational Welch's *t*‐test with Euclidian distance measures which is robust to unequal variances. All tests were run on PRIMER+ PERMANOVA. Curve best fit and tests for normality and homoscedasticity and *t*‐tests were run on R.

## 3 RESULTS

### 3.1 Embryonic development at heatwave treatments

In larvae that were raised at the same temperature to which they were spawned (similar to the control or heatwave temperature, that is, 23, 25 and 26 for control, moderate and strong, respectively), the rate of progression through developmental stages was faster in the heatwave treatments (Table S3). After 6 h, individuals from strong heatwave‐exposed parents developed faster with lower mortality than those spawned in control conditions across all developmental temperatures (strong × control, *t* = 3.35, *p* = .001; Figure 1). At 24 h post‐spawning, the composition of developmental stages in all three treatments differed, with those spawned in strong heatwave conditions at more advanced stages with less mortality (strong × control, *t* = 3.11, *p* = .001; strong × moderate, *t* = 2.59, *p* = .005), followed by moderate heatwave and control spawned larvae (moderate × control, *t* = 6.50, *p* = .001). At 48 and 72 h, larvae in moderate and strong heatwave conditions had developed faster than larvae from normal summer conditions (control), with most individuals at heatwave conditions reaching the larval or metamorphosed stage while those from control conditions were still at the blastula stage. Finally, after 144 h (6 days) post‐fertilization, the majority of individuals from all treatments were at a similar developmental stage, with most individuals being metamorphosed juveniles (Figure 1).

### 3.2 Development across a temperature range

**FIGURE 2:**
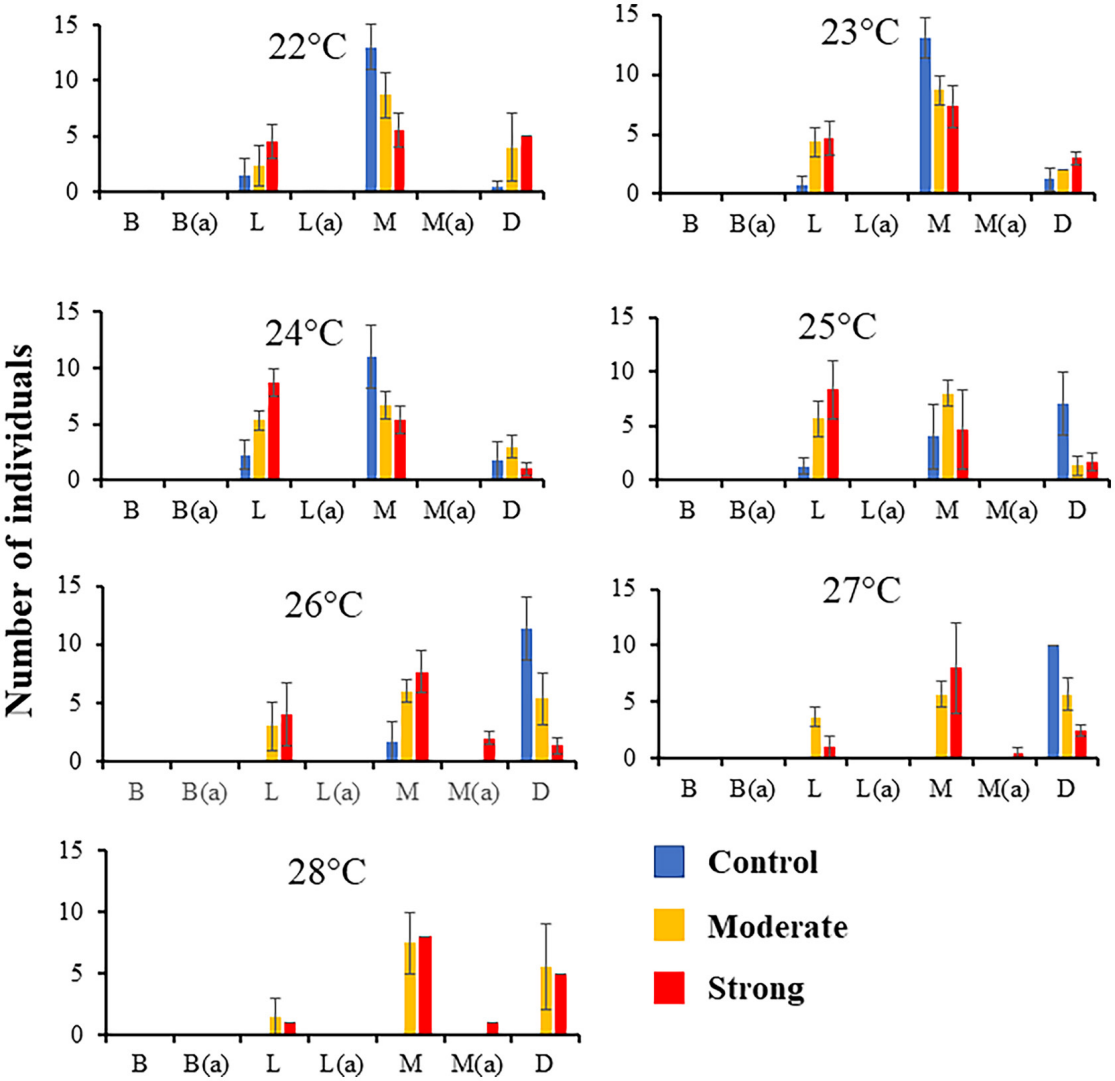
Larval developmental stages at 6 days post‐spawning. Individuals were sourced from parents conditioned in control (blue), moderate heatwave (yellow) or strong heatwave (red) conditions and reared at various temperatures (22–28°C). Graphs show the number of individuals at each developmental stage; blastula (B), larvae (L), metamorphosed (M) or dead (D). Individuals with abnormal development are represented by (a).

Individuals spawned from parents exposed to contemporary summer (control) conditions had different developmental population structures after 6 days (144 h; *F* = 5.58, *p* = 0.001, Table S4), depending on the temperature in which they were reared (Figure 2; Table S5). Individuals reared at 22, 23 and 24°C metamorphosed after 6 days while at higher temperatures (>25°C), fewer individuals metamorphosed and mortality became more prominent (Figure 2). Individuals originating from moderate and strong heatwave parents followed similar trends across all temperatures (22–28°C, moderate and strong *p* > .05; Figure 2; Table S5). The majority of individuals reached the larval or metamorphosed stage whilst fewer individuals died (Figure 2). Finally, some offspring of the strong heatwave‐exposed parents developed abnormalities at metamorphosis. Of those that reached metamorphosis at 26°C, at 27 and 28°C, there was 21%, 6% and 11% abnormal juveniles, respectively (Figure 2).

### 3.3 Juvenile size

**FIGURE 3:**
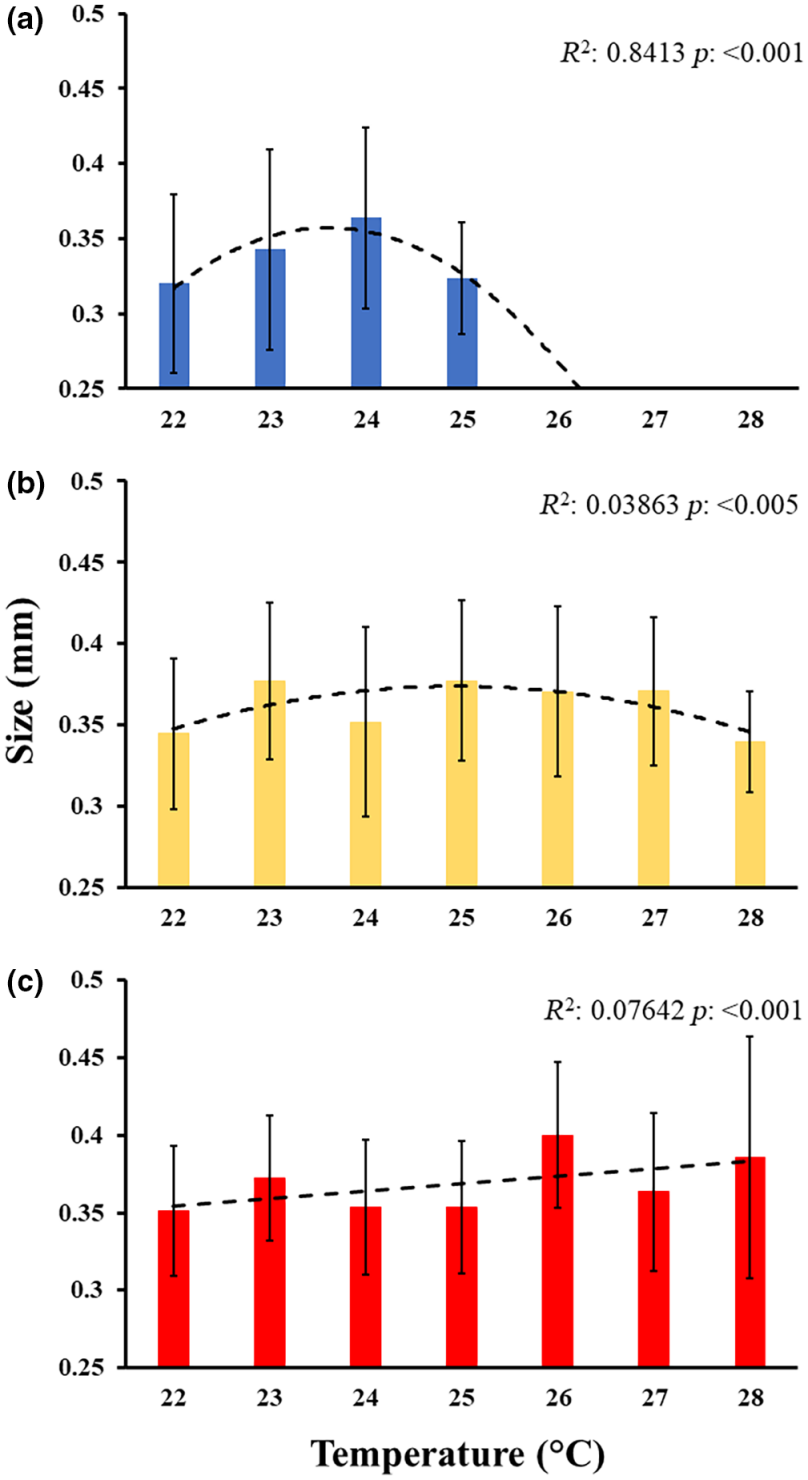
Juvenile size (mean ± *SD*) at 14 days post‐fertilization. Juveniles were produced from parents conditioned in (a) control (blue), (b) moderate heatwave (yellow) or (c) strong heatwave (red) conditions and reared at different temperatures (22–28°C). Trend lines represent curve of best fit (based on *R* ^2^ and significance of fit) following model estimation (quadratic: a and b; or linear: c).

After 14 days, juveniles generated from the heatwave‐conditioned parents showed dissimilar growth trends across the temperature gradient (moderate heatwave *F* = 3.28, *p* = .003; strong heatwave, *F* = 6.19, *p =* .001; Figure 3; Table S4). Juveniles of the control summer condition parents increased in size from 22 to 24°C, then decreased at 25°C and there was 100% mortality at temperatures above 25°C (Figure 3a; Table S6). The size of the juveniles of the moderate heatwave‐exposed parents increased from 22 to 25°C then decreased towards 28°C (Figure 3b; Table S6). In contrast, the size of juveniles from the strong heatwave‐exposed parents increased linearly from 22 to 28°C (Figure 3c; Table S6). Overall, juveniles from moderate heatwave‐exposed parents were able to maintain both size and survival throughout all temperatures while those from strong heatwave‐exposed parents grew bigger with increasing temperature, but also had higher mortality at high temperatures by the end of the experiment.

### 3.4 Survivorship across a thermal gradient

**FIGURE 4:**
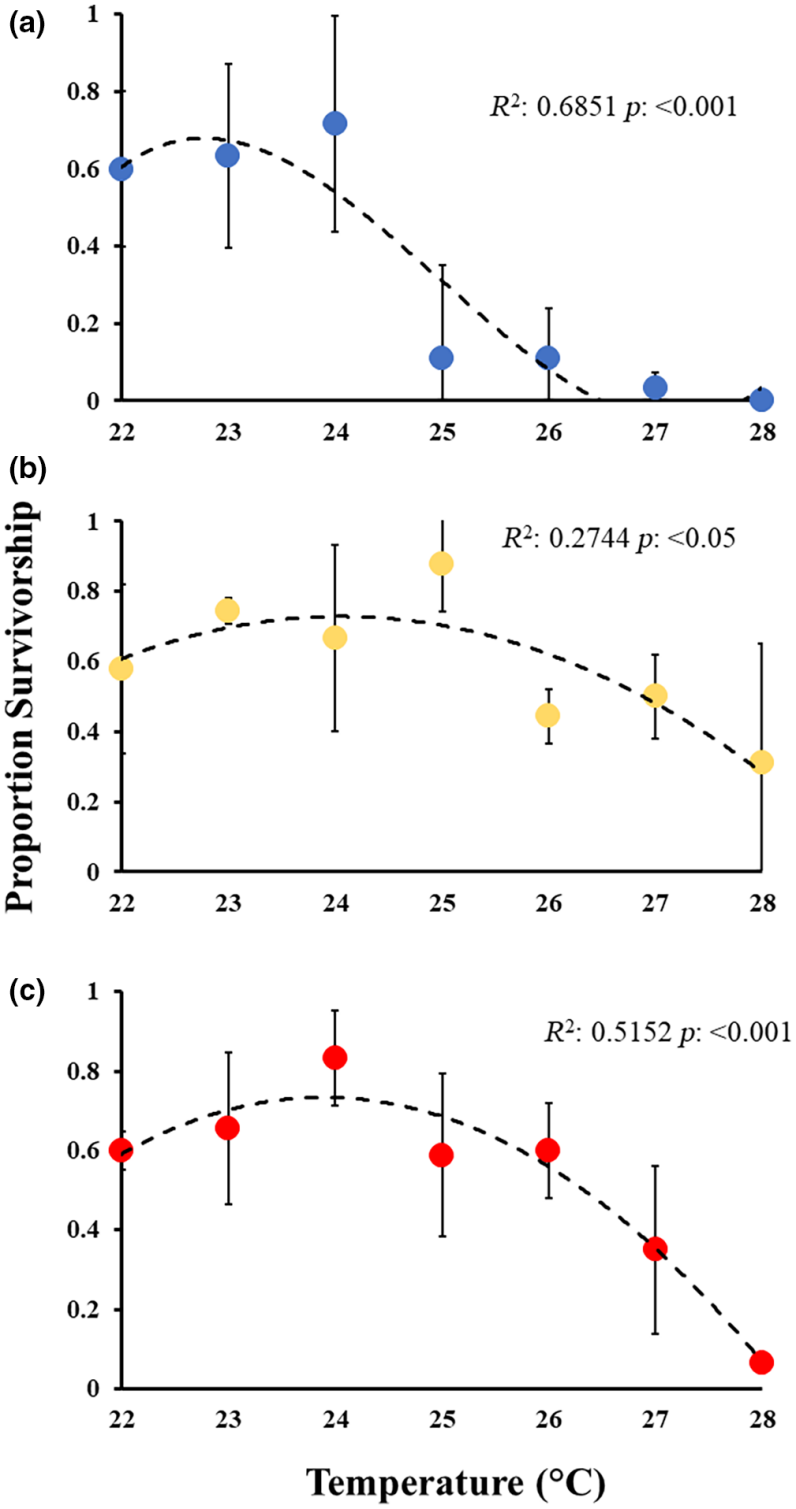
Proportion of larval survivorship (mean ± *SD*) at 6 days post‐fertilization. Larvae were hatched in (a) control (blue), (b) moderate heatwave (yellow) or (c) strong heatwave (red) conditions and reared at different temperatures (22–28°C). Trend lines represent curve of best fit based on *R* ^2^ and significance of fit.

The survival of larvae to metamorphosis (6 days) when exposed to temperatures from 22 to 28°C depended upon the heatwave conditions that their parents were exposed to. Larvae from control summer‐conditioned parents (23°C) had high survivorship from 22 to 24°C but this decreased drastically above 24°C (*F* = 11.26, *p* = .001; Figure 4; Table S4). More than 50% of the individuals from the moderate heatwave‐exposed parents (25°C) survived at all temperatures, except for the hottest rearing temperature (28°C), with survival increasing from 22 to 25°C (60%–80%, respectively) then decreasing towards 28°C (~40%; Figure 4; Table S7). Finally, larvae from strong heatwave‐conditioned parents (26°C) had high survival from 22 to 26°C (60%–80%, respectively) followed by drastic decrease at 27°C (~40%) and 28°C (<5%; *F* = 3.67, *p* = .04; Figure 4c; Table S7).

### 3.5 Juvenile respiration at heatwave temperatures

**FIGURE 5:**
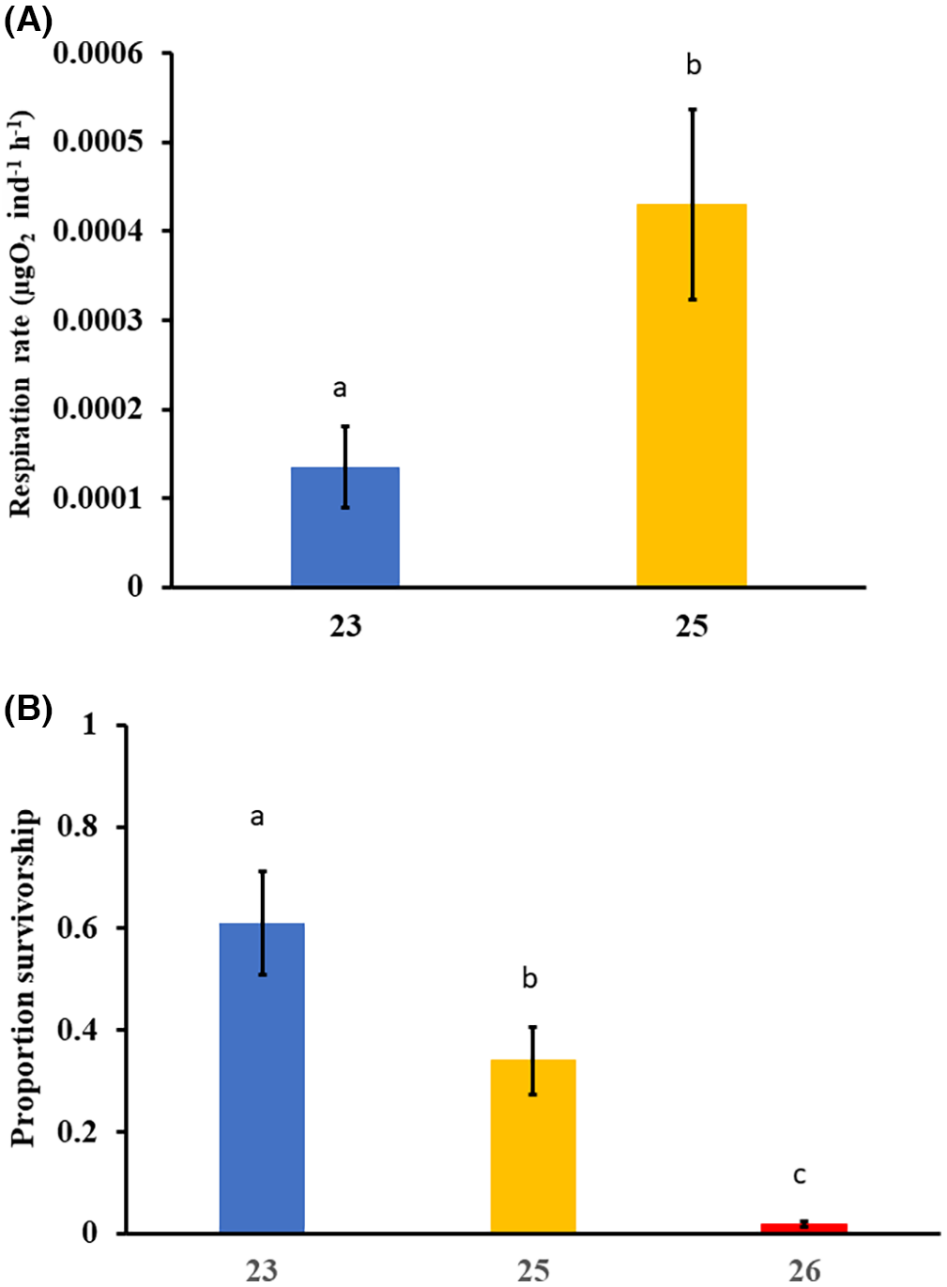
(A) Respiration rates (mean ± *SD*) of juveniles at 14‐day old reared at control (23°C) and moderate heatwave (25°C) conditions. Progeny were spawned from adults exposed to control (23°C) and moderate heatwave (25°C) conditions for 10 days prior to spawning. There were insufficient juveniles surviving from the strong heatwave (26°C) treatment adults to measure respiration. (B) Proportion of juvenile survivorship at day 14 post‐fertilization. Different letters above bars show significant differences.

Respiration rate of the juveniles (14 days post‐fertilization) generated from the adults exposed to moderate heatwave conditions (25°C) was approximately three times higher than juveniles from the summer control adults (23°C) when developed at their respective heatwave treatment temperatures (*t* = −9.94, *p* < .001; Figure 5). However, this higher respiration rate was associated with approximately double the mortality at moderate (25°C) than control summer (23°C) temperatures (Figure 5B). Respiration rates were not measured in juveniles from strong heatwave conditions because extremely low survival (<2%) meant that there were not enough individuals left after 14 days to gain meaningful measurements (Figure 5B).

## 4 DISCUSSION

The rate at which global temperatures are increasing due to anthropogenic climate change is resulting in longer, more frequent and more intense MHW events. From 1925 to 2016, the global frequency and duration of MHWs increased by 34% and 17%, respectively (Oliver et al., 2018), with models projecting a 41‐fold increase in the probability of MHWs by the end of the twenty‐first century, lasting on average 112 days at intensities of +2.5°C above current temperatures (Frölicher et al., 2018). With the increased occurrence of extreme events having devastating effects on marine organisms (Oliver et al., 2021), there has been substantial effort put towards understanding whether exposure of adults to increased temperature (heat hardening) can result in the inheritance of protective mechanisms for their offspring. Here, we found that exposure of adult urchins to heatwave temperatures prior to spawning did have positive effects on the growth rate and eventual survival of their offspring, but only as long as temperatures did not remain at heatwave levels into the settlement stages. Offspring from heatwave‐exposed parents had greater survival at higher temperatures in the first week of development, indicating potential protective carryover effects from adult to larva and an increased likelihood of surviving the projected increasing durations of MHWs (Holbrook et al., 2019; Smale et al., 2019). Other marine invertebrates have demonstrated shifts in the tolerance baselines of progeny following parental exposure to high temperatures. The thermal response of the F1 generation of the amphipod *Sunamphitoe parmerong* exhibits a shift in the upper lethal temperature when brooding females are acclimated to increased temperatures, with higher survival time at elevated temperatures compared to unacclimated individuals, indicating developmental plasticity (Campbell et al., 2020).

There were also carryover effects from larvae to juvenile; progeny of the strong heatwave‐exposed adults produced larger juveniles which reached settlement stages faster than any other treatment when developed under higher temperatures, indicating potential cross‐generational impacts of exposure to heatwave conditions during pre‐spawning gonad development. However, this enhanced early development was associated with a substantial cost—increased mortality by the time they reached the later juvenile stage. Other marine invertebrates have exhibited negative carryover effects during the developmental stages of following generations. Larvae to juvenile development of the starfish *Parvulastra exigua* was faster when exposed to MHW temperatures; however, survival rates decreased from 1 to 5 days post‐fertilization (Balogh & Byrne, 2020). Similar to the finding of our study, *Heliocidaris erythrogramma* juvenile tolerance has been shown to be plastic throughout development, with higher upper successful metamorphosis temperatures of 25.9°C by day 7 of development (+5°C above higher ambient conditions in the region), but by day 10 the juvenile tolerance narrowed back down to local range (16–19°C; Gall et al., 2021). Therefore, while this heat priming of the parents indicated that there are maternal factors passed onto the next generation which facilitate growth and survival in early benthic development, ultimately, this may not be efficient to ensure continued survival into the more sensitive later juvenile stages when most maternal reserves are gone (Byrne & Sewell, 2019; Emlet & Hoegh‐Guldberg, 1997; Falkner et al., 2015). The juveniles succumbed to mortality if heatwave conditions remained, showing the importance of a return to ambient conditions within thermal tolerance limits.

Cross‐generational plasticity is regarded as the non‐genetic inheritance of tolerance after adult exposure to the following generation (F0 to F1; Byrne et al., 2019). Whether any legacy is passed on to F1 generations is highly dependent on exposure time of the adults, and how much of gametogenic process occurred during stress exposure (Dupont et al., 2013; Suckling et al., 2015). For example, adults which are acclimated to ocean acidification and warming throughout gonadogenesis, and germ cell differentiation produce more resilient offspring to these stressors, but with a trade‐off of the reduction in overall size of the larvae (Karelitz, Lamare, Mos, et al., 2019). Similarly, paternal exposure to MHW temperatures in the purple sea urchin *Strongylocentrotus purpuratus* produced a negative impact on the fertilization success, whereas the temperature at which fertilization occurred had no impact on the fertilization itself, suggesting a hindering of the ability of sperm to reach the eggs and not abnormal development thereafter (Leach et al., 2021). Although the adults used in this study were already mature with developed gonads, which precludes a non‐genetic effect on the offspring (Byrne et al., 2019), the trade‐offs were evident with the alterations in size and development rates of the offspring. As typical of sea urchins, and marine invertebrates in general (Byrne, 2011; Hardy & Byrne, 2014), temperature increased the progression of the *H. erythrogramma* larvae through developmental stages. Only when strong conditions were sustained did the cross‐generational legacy of heat stressed parents result in eventual high mortality. This demonstrates the importance of assessing the impacts of heatwaves across the pelagic—benthic transition, as the positive parental carryover effects may only persist into later developmental stages should the environmental conditions not deviate from contemporary conditions for prolonged periods.

Physiological stress responses are exhibited as a short‐term mechanism for improving survival during events such as thermally stressful heatwaves (Dhabhar, 2014). When the stress responses are particularly intense, however, the energy required to maintain biological function can be incredibly costly (Caputi et al., 2016) causing changes to the biology of the organism and possible energetic trade‐offs (Fitzgerald‐Dehoog et al., 2012). Trade‐offs may be more immediate, such as reduced feeding and mass mortality events (Caputi et al., 2016; Chandrapavan et al., 2019; Leung et al., 2017) or cause knock‐on effects across generations (Byrne et al., 2009; Fitzgerald‐Dehoog et al., 2012; Karelitz, Lamare, Mos, et al., 2019). For example, adult *Heliocidaris erythrogramma* display higher metabolic rates in response to heatwave temperatures, which is not matched with concurrent increase in food consumption or faecal production, resulting in a latent decline in health and eventual mortality (Minuti et al., 2021). Elevated metabolic rates in larvae can also lead to increased mortality (Karelitz, Lamare, Patel, et al., 2019), generally because of depletion of energy stores (Leung et al., 2017; Sokolova, 2013). Therefore, coping mechanisms are time‐limited and typically only employed during short periods of adverse conditions. If the environmental stress (such as MHWs) occurs during spawning periods and continue throughout the larval development, these short‐term physiological responses may subsequently lead to deleterious effects for the continual growth and ultimate survival of next generation. If, however, carryover effects lead to increased function and survival under normally stressful temperatures, then larvae spawned during these extreme events may be more physiologically able to survive until conditions return to normal.

Physical trade‐offs in invertebrate offspring, such as reduction in size and increased incidence of morphological abnormalities, are often a direct effect of adult pre‐exposure to stress due to the influence that parental thermal history has on offspring phenotype (Byrne & Hernández, 2020; Karelitz, Lamare, Mos, et al., 2019; Lamare et al., 2014; Uthicke et al., 2013). For example, adult *Strongylocentrotus intermedius* urchins that were pre‐exposed to elevated seawater temperature produced offspring which had reduced larval length, width, post‐oral arm length/body rod length as well as increased percentage of abnormal larvae (Zhao et al., 2018). The tropical urchin *Toxopneustes rosseus* produced embryos with more than 90% developmental abnormalities and subsequent high mortality rates after exposure to elevated temperatures (Margarita Mejía‐Gutiérrez et al., 2019). Other echinoderms have also demonstrated physical trade‐offs in response to prior thermal stress regimes. When exposed to elevated temperatures during the developmental stage from metamorphosis to juvenile, the starfish *P. exigua* had increased incidences of perturbed body symmetry in response to the acute thermal stress and were overall smaller than the juveniles reared in cooler treatments (Balogh & Byrne, 2020). Early embryonic stages in the development of crown‐of‐thorns sea star (*Acanthaster* sp.) exhibit increasingly apparent rates of abnormality in temperatures above 28.7°C, reaching 100% at 33°C, just 1°C higher than the optimal thermal window for this species (~25–32°C; Lamare et al., 2014). Thus, the physical trade‐offs that occur during embryonic and larval development as a response to elevated seawater temperatures show that physiological malfunction may occur at any developmental stage with flow‐on effects to overall performance or survival of individuals. While exposure of *H. erythrogramma* larvae to higher temperatures immediately following spawning at summer temperatures led to higher proportions of abnormalities and mortality within the population, this was offset in the short term if the parents were pre‐exposed to elevated temperatures. Therefore, the timing of MHWs will be paramount; if the heatwave affects adults before or during spawning but subsides within the first few days after fertilization, then larvae will have maternal protection and survive. In contrast, if the heatwave manifests after spawning, then larval mortality will be high, potentially at the cost of the population.

When exposed to elevated temperatures over the longer term, adult *H. erythrogramma* show a range of physiological responses such as the production of heat shock proteins (Nguyen et al., 2013) or metabolic depression to alleviate stress at high temperatures (Harianto et al., 2018, 2021; Minuti et al., 2021). Larvae are known to be able to withstand considerable thermal change during the earliest stages of development, becoming more susceptible to temperature induced damage as they reach early adult phases (Dworjanyn & Byrne, 2018; Foo et al., 2016). The different parental effects on developmental stages of planktonic larvae highlight the need to assess the sensitivity to thermal stress across life stages, from adult exposure and spawning conditions, and through the whole of the developmental process. Higher larval metabolic rates may suggest that there is an increase in resources used in the earlier stages of development after the parents have been exposed to harsh conditions, to prepare the progeny for unfavourable circumstances during early life (Karelitz, Lamare, Mos, et al., 2019). Yet, elevated metabolic rates in response to environmental stress can ultimately threaten the continued development of larvae by decreasing scope for growth and delaying development by reallocation of energy available for growth to metabolic maintenance (Stumpp et al., 2011). Here, it is evident that the exposure of adult urchins to heatwaves, even after the gametogenic process has already occurred, can result in faster development and a generation of larger juveniles. Therefore, the carryover effects from heat hardening of parents could increase survival and development of larvae spawned during heatwaves. Once settled, however, there was little evidence to suggest that any resilience had been carried through to the post‐settlement stage if heatwave temperatures continued, and thus the trade‐off manifests and mortality ensued, preventing development of the next generation and a reduction in reproductive output for the population with prolonged extreme thermal events.

## CONFLICT OF INTEREST

The authors have no conflict of interest to declare.

## Supporting information


Figure S1
Table S1
Table S2
Table S3
Table S4
Table S5
Table S6
Table S7

## Data Availability

Data are publicly available through the University of Hong Kong Libraries DataHub (https://datahub.hku.hk/, https://doi.org/10.25442/hku.20105366).
