# Supplementary material for: Live‐fast‐die‐young: Carryover effects of heatwave‐exposed adult urchins on the development of the next generation
Source: Glob Chang Biol. 2022 Aug 3;28(19):5781–92. doi: 10.1111/gcb.16339 (PMC9805142; doi:10.1111/gcb.16339)
Supplement: Supplementary file 1 — Figure S1 Table S1 Table S2 Table S3 Table S4 Table S5 Table S6 Table S7 [file GCB-28-5781-s001.docx]

**Supporting Information**

# Live-fast-die-young: carry-over effects of heatwave exposed adult urchins on the development of the next generation

Jay J. Minuti, Maria Byrne, Hamish Campbell, Deevesh A. Hemraj, Bayden D. Russell

**Supplementary Figure 1:** Schematic diagram of the experimental design and time-points of data collection.

**Supplementary Table 1**: Summary table of normality and homoscedasticity tests on larval size and survival data. Control, moderate, and strong represent the marine heatwave conditions in which larvae were spawned.

| Data | Test | Temperature | df | p-value |
| --- | --- | --- | --- | --- |
| Size control | Shapiro-Wilk | 22 | 31 | 0.690 |
|  |  | 23 | 34 | 0.473 |
|  |  | 24 | 36 | 0.059 |
|  |  | 25 | 18 | 0.702 |
|  | Bartlett’s test |  | 6 | <0.001 |
|  |  |  |  |  |
| Size moderate | Shapiro-Wilk | 22 | 40 | 0.206 |
|  |  | 23 | 60 | 0.051 |
|  |  | 24 | 9 | 0.723 |
|  |  | 25 | 40 | 0.525 |
|  |  | 26 | 51 | 0.693 |
|  |  | 27 | 35 | 0.091 |
|  |  | 28 | 11 | 0.462 |
|  | Bartlett’s test |  | 6 | >0.05 |
|  |  |  |  |  |
| Size strong | Shapiro-Wilk | 22 | 32 | 0.740 |
|  |  | 23 | 43 | 0.758 |
|  |  | 24 | 52 | 0.006 |
|  |  | 25 | 57 | 0.955 |
|  |  | 26 | 52 | 0.462 |
|  |  | 27 | 20 | 0.357 |
|  |  | 28 | 14 | 0.024 |
|  | Bartlett’s test |  | 6 | <0.05 |
|  |  |  |  |  |
| Survival control | Shapiro-Wilk | 22 | 4 | 0.001 |
|  |  | 23 | 4 | 0.457 |
|  |  | 24 | 4 | 0.312 |
|  |  | 26 | 4 | 0.250 |
|  |  | 27 | 4 | 0.024 |
|  | Bartlett’s test |  | 6 | <0.001 |
|  |  |  |  |  |
| Survival moderate | Shapiro-Wilk | 22 | 3 | 0.132 |
|  |  | 23 | 3 | 0.000 |
|  |  | 24 | 3 | 0.363 |
|  |  | 25 | 3 | 0.726 |
|  |  | 26 | 3 | 0.000 |
|  |  | 27 | 3 | 0.537 |
|  |  | 28 | 3 | 0.281 |
|  | Bartlett’s test |  | 6 | >0.05 |
|  |  |  |  |  |
| Survival strong | Shapiro-Wilk | 22 | 3 | 0.000 |
|  |  | 23 | 3 | 0.537 |
|  |  | 24 | 3 | 0.637 |
|  |  | 25 | 3 | 0.537 |
|  | Bartlett’s test |  | 6 | >0.05 |

**Supplementary Table 2**: Summary of best fit regressions representing size and survival probability over the temperature gradient. Control, moderate, and strong represent the marine heatwave conditions in which larvae were spawned.

|  | R^2^ | P-value | F-statictics | df | AIC | BIC |
| --- | --- | --- | --- | --- | --- | --- |
| Size |  |  |  |  |  |  |
| control | 0.8413 | <0.001 | 361.5 | 201 | -505.04 | -488.43 |
| moderate | 0.03863 | <0.005 | 4.282 | 242 | -785.21 | -767.69 |
| strong | 0.07642 | <0.001 | 12.13 | 267 | 1020.39 | 1034.78 |
| Survival |  |  |  |  |  |  |
| control | 0.6851 | <0.001 | 19.13 | 22 | -6.26 | 0.028 |
| moderate | 0.2744 | <0.05 | 3.521 | 17 | -0.19 | 5.04 |
| strong | 0.5152 | <0.05 | 6.667 | 13 | -8.68 | -4.51 |

**Supplementary Table 3:** PERMANOVA tests comparing the changes in larval population structure (blastula, larvae, metamorphosed or dead) at 6, 24, 48, 71 and 144 hours post spawning from either control summer temperature, moderate heatwave, or strong heatwave conditions. For pairwise comparisons, the more conservative p<0.01 is used for significance to account for potentially inflated error rates with multiple comparisons.

| Larval population | Spawned condition | Df | SS | MS | pseudo f | t | p-value |
| --- | --- | --- | --- | --- | --- | --- | --- |
|  |  |  |  |  |  |  |  |
| 6 hours | Main test | 2 | 85.301 | 42.65 | 6.4143 |  | 0.001 |
|  | *residual* | 60 | 398.95 | 6.6492 |  |  |  |
|  |  |  |  |  |  |  |  |
|  | Control x Moderate | 44 |  |  |  | 1.8157 | 0.062 |
|  | Control x Strong | 40 |  |  |  | 3.3513 | 0.001 |
|  | Moderate x Strong | 36 |  |  |  | 1.9865 | 0.02 |
|  |  |  |  |  |  |  |  |
| 24 hours | Main test | 2 | 2323.4 | 1161.7 | 20.123 |  | 0.001 |
|  | *residual* | 63 | 3636.9 | 57.728 |  |  |  |
|  |  |  |  |  |  |  |  |
|  | Control x Moderate | 47 |  |  |  | 6.4968 | 0.001 |
|  | Control x Strong | 43 |  |  |  | 3.1099 | 0.001 |
|  | Moderate x Strong | 36 |  |  |  | 2.5875 | 0.005 |
|  |  |  |  |  |  |  |  |
| 48 hours | Main test | 2 | 875.5 | 437.75 | 9.5926 |  | 0.001 |
|  | *residual* | 63 | 2875 | 45.634 |  |  |  |
|  |  |  |  |  |  |  |  |
|  | Control x Moderate | 47 |  |  |  | 3.6623 | 0.001 |
|  | Control x Strong | 43 |  |  |  | 2.8548 | 0.001 |
|  | Moderate x Strong | 36 |  |  |  | 1.4921 | 0.081 |
|  |  |  |  |  |  |  |  |
| 72 hours | Main test | 2 | 327.42 | 163.71 | 4.307 |  | 0.005 |
|  | *residual* | 63 | 2318.6 | 38.01 |  |  |  |
|  |  |  |  |  |  |  |  |
|  | Control x Moderate | 45 |  |  |  | 2.5858 | 0.002 |
|  | Control x Strong | 41 |  |  |  | 2.0933 | 0.01 |
|  | Moderate x Strong | 36 |  |  |  | 1.1319 | 0.264 |
|  |  |  |  |  |  |  |  |
| 144 hours | Main test | 2 | 234.59 | 117.3 | 2.7702 |  | 0.032 |
|  | *residual* |  | 2286.5 | 42.343 |  |  |  |
|  |  |  |  |  |  |  |  |
|  | Control x Moderate | 38 |  |  |  | 1.3523 | 0.162 |
|  | Control x Strong | 35 |  |  |  | 2.0465 | 0.027 |
|  | Moderate x Strong | 35 |  |  |  | 1.3023 | 0.182 |

**Supplementary Table 4**: PERMANOVA tests identifying differences in larval population structure, juvenile size and survival of eggs spawned in either control, moderate heatwave, or Strong heatwave after they were reared at different temperatures (22-28°C).

|  | Spawned condition | Df | SS | MS | Pseudo-F | P-value |
| --- | --- | --- | --- | --- | --- | --- |
| larval Population structure | Control (23°C) | 6 | 1170.4 | 195.07 | 5.5773 | 0.001 |
|  | *residual* | 21 | 734.5 | 34.976 |  |  |
|  |  |  |  |  |  |  |
|  | Moderate heatwave (25°C) | 6 | 136.38 | 22.73 | 0.89724 | 0.596 |
|  | *residual* | 14 | 354.67 | 25.33 |  |  |
|  |  |  |  |  |  |  |
|  | Strong heatwave (26°C) | 6 | 195.65 | 32.608 | 1.1091 | 0.394 |
|  | *residual* | 10 | 294 | 29.4 |  |  |
|  |  |  |  |  |  |  |
| Juvenile size | Control (23°C) | 3 | 0.03759 | 0.01253 | 3.5596 | 0.021 |
|  | *residual* | 115 | 0.4048 | 0.00352 |  |  |
|  |  |  |  |  |  |  |
|  | Moderate heatwave (25°C) | 6 | 0.045483 | 0.0075805 | 3.2764 | 0.003 |
|  | *residual* | 239 | 0.55297 | 0.0023137 |  |  |
|  |  |  |  |  |  |  |
|  | Strong heatwave (26°C) | 6 | 0.079606 | 0.013268 | 6.1884 | 0.001 |
|  | *residual* | 263 | 0.56386 | 0.0021439 |  |  |
|  |  |  |  |  |  |  |
| Survival | Control (23°C) | 6 | 2.4315 | 0.40525 | 11.255 | 0.001 |
|  | *residual* | 21 | 0.75611 | 0.036005 |  |  |
|  |  |  |  |  |  |  |
|  | Moderate heatwave (25°C) | 6 | 0.65926 | 0.10988 | 2.7075 | 0.068 |
|  | *residual* | 14 | 0.56815 | 0.040582 |  |  |
|  |  |  |  |  |  |  |
|  | Strong heatwave (26°C) | 6 | 0.58005 | 0.096676 | 3.6894 | 0.036 |
|  | *residual* | 10 | 0.26204 | 0.026204 |  |  |

**Supplementary Table 5**: Pairwise PERMANOVA tests comparing the larval population structure at 6 days post spawning in either control, moderate heatwave or strong heatwave conditions and reared at various temperatures (22-28°C).

| Treatment | Pairwise temperature comparison (°C) | Df | t | p-value |
| --- | --- | --- | --- | --- |
| Control 23°C | 22- 23 | 6 | 0.32287 | 0.71 |
|  | 22- 24 | 6 | 0.56347 | 0.73 |
|  | 22- 25 | 6 | 2.2406 | 0.061 |
|  | 22- 26 | 6 | 3.2113 | 0.019 |
|  | 22- 27 | 6 | 3.7074 | 0.023 |
|  | 22- 28 | 6 | 5.1366 | 0.035 |
|  | 23- 24 | 6 | 0.61684 | 0.702 |
|  | 23- 25 | 6 | 2.2613 | 0.049 |
|  | 23- 26 | 6 | 3.2955 | 0.033 |
|  | 23- 27 | 6 | 4.029 | 0.023 |
|  | 23- 28 | 6 | 6.3146 | 0.021 |
|  | 24- 25 | 6 | 1.5855 | 0.134 |
|  | 24- 26 | 6 | 2.3624 | 0.092 |
|  | 24- 27 | 6 | 2.5787 | 0.042 |
|  | 24- 28 | 6 | 3.1772 | 0.032 |
|  | 25-26 | 6 | 0.60212 | 0.733 |
|  | 25- 27 | 6 | 1.2471 | 0.26 |
|  | 25- 28 | 6 | 1.9197 | 0.028 |
|  | 26- 27 | 6 | 1.3857 | 0.257 |
|  | 26- 28 | 6 | 2.3547 | 0.138 |
|  | 27- 28 |  | 1 | 1 |
|  |  |  |  |  |
| Moderate heatwave 25°C | 22- 23 | 5 | 0.636 | 0.911 |
|  | 22- 24 | 5 | 0.83435 | 0.589 |
|  | 22- 25 | 5 | 0.92582 | 0.497 |
|  | 22- 26 | 5 | 0.58794 | 0.791 |
|  | 22- 27 | 5 | 0.79921 | 0.521 |
|  | 22- 28 | 5 | 0.67487 | 0.798 |
|  | 23- 24 | 4 | 0.99087 | 0.529 |
|  | 23- 25 | 4 | 0.58554 | 0.795 |
|  | 23- 26 | 4 | 1.2403 | 0.39 |
|  | 23- 27 | 4 | 1.7802 | 0.21 |
|  | 23- 28 | 4 | 1.1789 | 0.369 |
|  | 24- 25 | 4 | 0.75851 | 0.717 |
|  | 24- 26 | 4 | 0.92195 | 0.429 |
|  | 24- 27 | 4 | 1.2005 | 0.297 |
|  | 24- 28 | 4 | 1.0488 | 0.384 |
|  | 25- 26 | 4 | 1.3444 | 0.331 |
|  | 25- 27 | 4 | 1.7494 | 0.188 |
|  | 25- 28 | 4 | 1.293 | 0.208 |
|  | 26- 27 | 4 | 0.21483 | 1 |
|  | 26- 28 | 4 | 0.53783 | 0.907 |
|  | 27- 28 | 4 | 0.73984 | 0.704 |
|  |  |  |  |  |
| STRONG heatwave 26°C | 22- 23 | 3 | 0.79268 | 0.495 |
|  | 22- 24 | 3 | 2.0597 | 0.106 |
|  | 22- 25 | 3 | 0.82845 | 0.471 |
|  | 22- 26 | 3 | 1.0276 | 0.395 |
|  | 22- 27 | 2 | 1.066 | 0.674 |
|  | 22- 28 | 1 | 1.2019 | 0.686 |
|  | 23- 24 | 4 | 1.6535 | 0.297 |
|  | 23- 25 | 4 | 0.90576 | 0.512 |
|  | 23- 26 | 4 | 0.66112 | 0.593 |
|  | 23- 27 | 3 | 0.87066 | 0.504 |
|  | 23- 28 | 2 | 0.92195 | 0.522 |
|  | 24- 25 | 4 | 0.20045 | 0.875 |
|  | 24- 26 | 4 | 1.4728 | 0.295 |
|  | 24- 27 | 3 | 2.1287 | 0.095 |
|  | 24- 28 | 2 | 2.5359 | 0.24 |
|  | 25- 26 | 4 | 0.98468 | 0.272 |
|  | 25- 27 | 3 | 1.1985 | 0.301 |
|  | 25- 28 | 2 | 0.94258 | 0.755 |
|  | 26- 27 | 3 | 0.669 | 0.68 |
|  | 26- 28 | 2 | 0.7244 | 0.732 |
|  | 27- 28 | 1 | 0.35187 | 1 |

**Supplementary Table 6**: Pairwise PERMANOVA tests comparing the size of juveniles spawned in either control, moderate heatwave or strong heatwave conditions and reared at various temperatures (22-28°C).

| Treatment | Pairwise temperature comparison (°C) | Df | t | p-value |
| --- | --- | --- | --- | --- |
| Control 23°C | 22-23 | 63 | 1.4378 | 0.162 |
|  | 22-24 | 65 | 2.9779 | 0.006 |
|  | 22-25 | 47 | 0.24137 | 0.8 |
|  | 23-24 | 68 | 1.3816 | 0.183 |
|  | 23-25 | 50 | 1.1062 | 0.295 |
|  | 24-25 | 52 | 2.5643 | 0.014 |
|  |  |  |  |  |
| Moderate heatwave 25°C | 22-23 | 98 | 3.6212 | 0.001 |
|  | 22-24 | 47 | 0.53648 | 0.621 |
|  | 22-25 | 78 | 3.2973 | 0.003 |
|  | 22-26 | 89 | 2.6905 | 0.012 |
|  | 22-27 | 73 | 2.7045 | 0.01 |
|  | 22-28 | 49 | 0.1947 | 0.841 |
|  | 23-24 | 67 | 1.4273 | 0.166 |
|  | 23-25 | 98 | 0.018562 | 0.99 |
|  | 23-26 | 109 | 0.68318 | 0.493 |
|  | 23-27 | 93 | 0.61958 | 0.544 |
|  | 23-28 | 69 | 2.4826 | 0.021 |
|  | 24-25 | 47 | 1.3487 | 0.174 |
|  | 24-26 | 58 | 0.9655 | 0.301 |
|  | 24-27 | 42 | 1.0473 | 0.283 |
|  | 24-28 | 18 | 0.59906 | 0.536 |
|  | 25-26 | 89 | 0.62076 | 0.55 |
|  | 25-27 | 73 | 0.58005 | 0.549 |
|  | 25-28 | 49 | 2.3907 | 0.015 |
|  | 26-27 | 84 | 0.028026 | 0.976 |
|  | 26-28 | 60 | 1.8698 | 0.067 |
|  | 27-28 | 44 | 2.1034 | 0.038 |
|  |  |  |  |  |
| STRONG heatwave 26°C | 22-23 | 73 | 2.222 | 0.028 |
|  | 22-24 | 82 | 0.29271 | 0.751 |
|  | 22-25 | 87 | 1.2503 | 0.231 |
|  | 22-26 | 82 | 4.8145 | 0.001 |
|  | 22-27 | 50 | 0.96201 | 0.32 |
|  | 22-28 | 44 | 1.9841 | 0.06 |
|  | 23-24 | 93 | 2.1278 | 0.04 |
|  | 23-25 | 98 | 1.1348 | 0.274 |
|  | 23-26 | 93 | 3.0394 | 0.005 |
|  | 23-27 | 61 | 0.74126 | 0.451 |
|  | 23-28 | 55 | 0.86432 | 0.395 |
|  | 24-25 | 107 | 1.0772 | 0.307 |
|  | 24-26 | 102 | 5.1864 | 0.001 |
|  | 24-27 | 70 | 0.80543 | 0.437 |
|  | 24-28 | 64 | 2.0431 | 0.039 |
|  | 25-26 | 107 | 4.3267 | 0.001 |
|  | 25-27 | 75 | 0.065344 | 0.947 |
|  | 25-28 | 69 | 1.5255 | 0.132 |
|  | 26-27 | 70 | 2.8784 | 0.005 |
|  | 26-28 | 64 | 0.84331 | 0.405 |
|  | 27-28 | 32 | 1.0233 | 0.32 |

**Supplementary Table 7**: Pairwise PERMANOVA tests comparing the survival of juveniles spawned in either control, moderate heatwave or strong heatwave conditions and reared at various temperatures (22-28°C).

| Treatment | Pairwise temperature comparison (°C) | Df | t | p-value |
| --- | --- | --- | --- | --- |
| Control 23°C | 22- 23 | 6 | 0.21401 | 1 |
|  | 22- 24 | 6 | 0.68097 | 0.62 |
|  | 22- 25 | 6 | 3.1285 | 0.026 |
|  | 22- 26 | 6 | 4.1074 | 0.036 |
|  | 22- 27 | 6 | 5.5646 | 0.028 |
|  | 22- 28 | 6 | 6 | 0.031 |
|  | 23- 24 | 6 | 0.45455 | 0.609 |
|  | 23- 25 | 6 | 3.0851 | 0.054 |
|  | 23- 26 | 6 | 3.8507 | 0.038 |
|  | 23- 27 | 6 | 4.9606 | 0.021 |
|  | 23- 28 | 6 | 5.3038 | 0.037 |
|  | 24- 25 | 6 | 3.2967 | 0.064 |
|  | 24- 26 | 6 | 3.9532 | 0.023 |
|  | 24- 27 | 6 | 4.8658 | 0.031 |
|  | 24- 28 | 6 | 5.1518 | 0.027 |
|  | 25- 27 | 6 | 0.61096 | 0.722 |
|  | 25- 28 | 6 | 0.89355 | 0.704 |
|  | 26- 27 | 6 | 1.0941 | 0.412 |
|  | 26- 28 | 6 | 1.6466 | 0.402 |
|  | 27- 28 | 6 | 1.7321 | 0.427 |
|  |  |  |  |  |
| Moderate heatwave 25°C | 22- 23 | 5 | 1.6271 | 0.232 |
|  | 22- 24 | 5 | 0.24007 | 0.691 |
|  | 22- 25 | 5 | 2.2313 | 0.122 |
|  | 22- 26 | 5 | 1.3027 | 0.288 |
|  | 22- 27 | 5 | 0.83293 | 0.399 |
|  | 22- 28 | 5 | 1.0531 | 0.355 |
|  | 23- 24 | 4 | 0.50387 | 1 |
|  | 23- 25 | 4 | 1.6483 | 0.308 |
|  | 23- 26 | 4 | 6.0374 | 0.108 |
|  | 23- 27 | 4 | 3.355 | 0.106 |
|  | 23- 28 | 4 | 2.1905 | 0.209 |
|  | 24- 25 | 4 | 1.2316 | 0.41 |
|  | 24- 26 | 4 | 1.3969 | 0.433 |
|  | 24- 27 | 4 | 0.9934 | 0.407 |
|  | 24- 28 | 4 | 1.4282 | 0.22 |
|  | 25- 26 | 4 | 4.8374 | 0.101 |
|  | 25- 27 | 4 | 3.6244 | 0.114 |
|  | 25- 28 | 4 | 2.6805 | 0.111 |
|  | 26- 27 | 4 | 0.6742 | 0.704 |
|  | 26- 28 | 4 | 0.66158 | 0.487 |
|  | 27- 28 | 4 | 0.9061 | 0.482 |
|  |  |  |  |  |
| STRONG heatwave 26°C | 22- 23 | 3 | 0.38162 | 1 |
|  | 22- 24 | 3 | 2.51 | 0.212 |
|  | 22- 25 | 3 | 0.072232 | 1 |
|  | 22- 26 | 3 | 1.12E-08 | 1 |
|  | 22- 27 | 2 | 1.627 | 0.327 |
|  | 22- 28 | 1 | 9.2376 | 0.338 |
|  | 23- 24 | 4 | 1.3571 | 0.289 |
|  | 23- 25 | 4 | 0.41208 | 0.71 |
|  | 23- 26 | 4 | 0.42409 | 0.627 |
|  | 23- 27 | 3 | 1.6801 | 0.309 |
|  | 23- 28 | 2 | 2.65 | 0.278 |
|  | 24- 25 | 4 | 1.7903 | 0.204 |
|  | 24- 26 | 4 | 2.3778 | 0.188 |
|  | 24- 27 | 3 | 3.3737 | 0.106 |
|  | 24- 28 | 2 | 5.5244 | 0.232 |
|  | 25- 26 | 4 | 0.081379 | 1 |
|  | 25- 27 | 3 | 1.2671 | 0.288 |
|  | 25- 28 | 2 | 2.2205 | 0.252 |
|  | 26- 27 | 3 | 1.745 | 0.291 |
|  | 26- 28 | 2 | 3.8431 | 0.255 |
|  | 27- 28 | 1 | 1.0906 | 0.677 |
